# Supplementary material for: Structural and Kinetic Characterization of the SpeG Spermidine/Spermine N-acetyltransferase from Methicillin-Resistant Staphylococcus aureus USA300
Source: Cells. 2023 Jul 12;12(14):1829. doi: 10.3390/cells12141829 (PMC10378331; doi:10.3390/cells12141829)
Supplement: Supplementary file 1 [file cells-12-01829-s001.zip › cells-2408519-supplementary.pdf]

SUPPLEMENTARY FIGURES

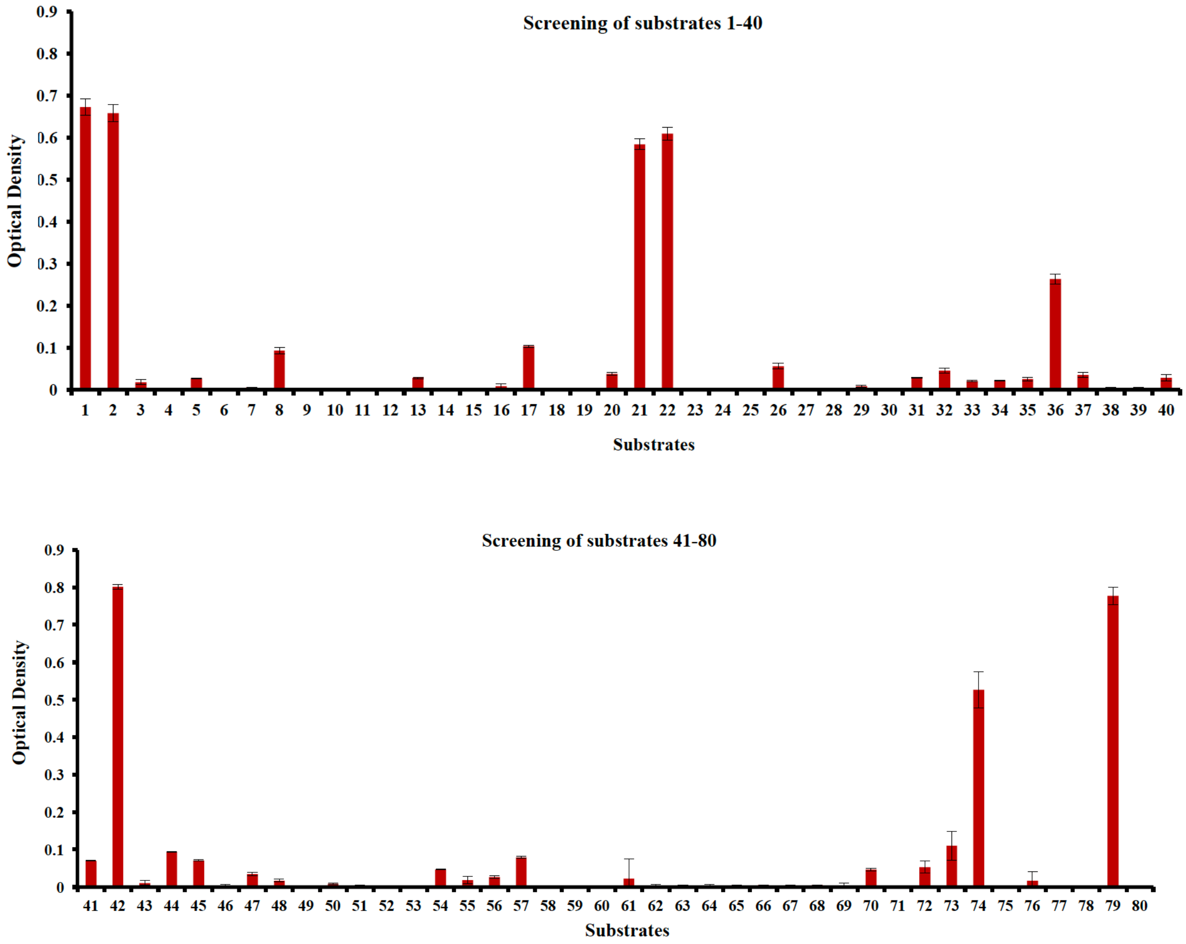

Figure S1. Broad-substrate screening of the 1-80 substrates for SaSpeG. SaSpeG showed activity toward 1: Spermine, 2: Spermidine, 21: Cadaverine, 22: Putrescine, 42: Agmatine, and 79: N-acetyl spermine. Details of the 1-80 compound names are indicated in **Table S1**.

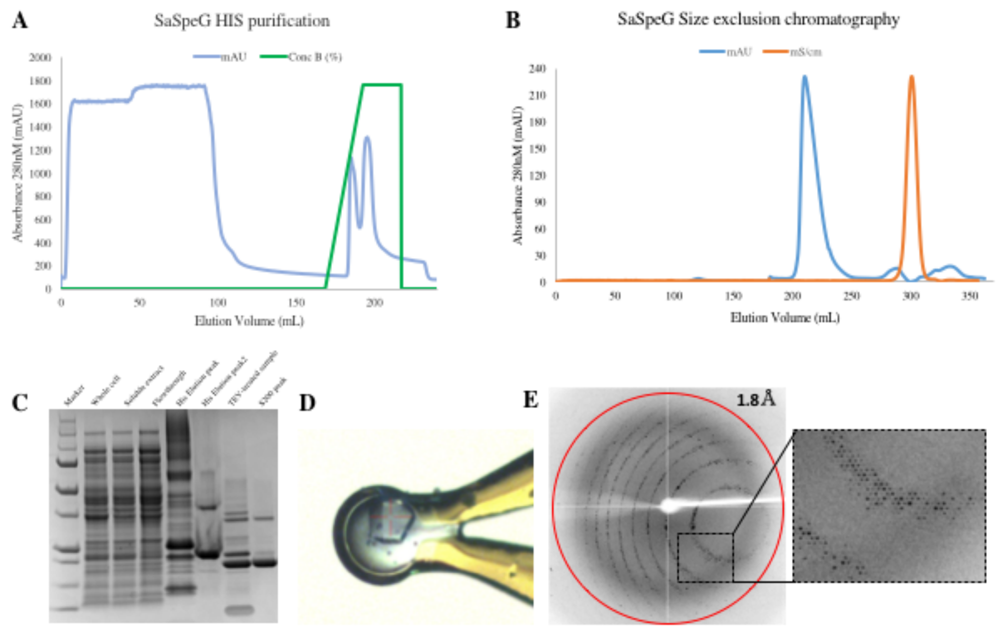

Figure S2: Purification of the *Sa*SpeG by affinity and size exclusion chromatography yielding >95% pure protein **A)** His-tag affinity and **B)** Size exclusion chromatography profiles with corresponding lane markers 4, 5, and 7 from C, showing a single, homogenous peak eluting from the size exclusion column at approximately molecular 70kDa **C)** SDS-PAGE analysis of samples through the purification process **1)** size marker **2)** whole cell bacterial lysate showing over-expressed SaSpeG **3)** soluble supernatant following centrifugation **4)** the flow-through of unbound proteins following loading of the supernatant onto the affinity column **5)** elution of His-tagged SaSpeG protein **6)** His-tag removal from SaSpeG by TEV protease **7)** SaSpeG following size exclusion chromatography. **D**) crystal of SaSpeG in a loop for diffraction **E)** X-ray diffraction image showing data collected at 1.8Å for the PDB ID 5IX3 structure.

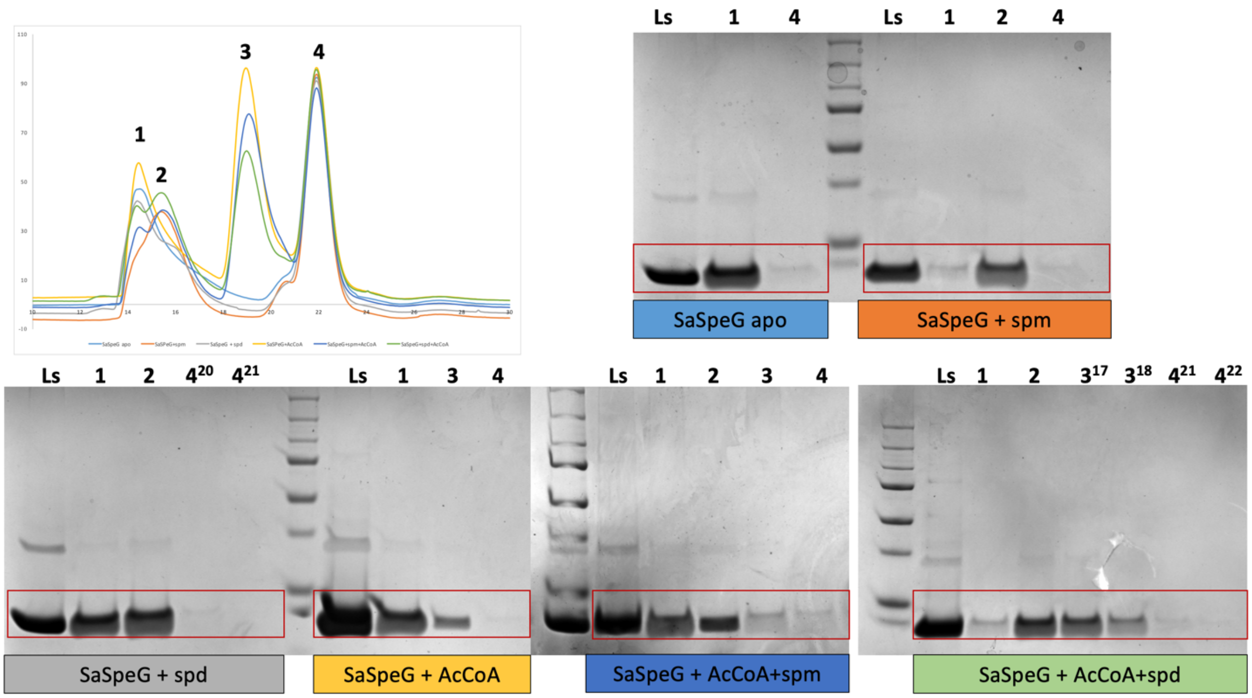

Figure S3. Analytical size exclusion chromatography and SDS-PAGE of SaSpeG. Comparison of size exclusion chromatograms of SaSpeG in apo form (light blue) and in presence of spermine (spm) (orange), spermidine (spd) (grey), AcCoA (yellow), spm and AcCoA (dark blue), and spd and AcCoA (green) showing presence of multiple assemblies in solution represented by peaks labelled 1, 2, 3 and 4 for different elution volumes. Samples were analyzed via SDS-PAGE and included Ls (sample prior to being loaded onto the column) and different fractions for each corresponding peak (1-4) as indicated on the chromatograms.

TABLES

Table S1: List of compounds screened in the broad-substrate screening assay.

**No.**	**Name of substrate**	**No.**	**Name of substrate**
**1**	Spermine	**41**	L-Asparagine
**2**	Spermidine trihydrochloride	**42**	Agmatine sulfate salt
**3**	Thialysine	**43**	L-Arginine
**4**	L-Lysine	**44**	L-Aspartic acid
**5**	Poly-L-lysine hydrobromide	**45**	L-Glutamic acid
**6**	L-serine	**46**	L-Phenylalanine
**7**	L-threonine	**47**	Kanamycin B sulfate salt
**8**	L-methionine	**48**	Apramycin sulfate salt
**9**	N-Acetyl-L-methionine	**49**	NADP
**10**	Neomycin trisulfate salt hydrate	**50**	Creatine
**11**	Gentamicin	**51**	Cytidine
**12**	Puromycin dihydrochloride	**52**	L-tyrosine
**13**	Chloramphenicol	**53**	L-valine
**14**	D-(+)-Glucosamine hydrochloride	**54**	antibiotic G418
**15**	D-Glucosamine 6-phosphate	**55**	Polymyxin B sulfate
**16**	Dopamine hydrochloride	**56**	Bacitracin
**17**	Epinephrine	**57**	Adenosine
**18**	Ethyl-4-aminobutyrate hydrochloride	**58**	Guanidine hydrochloride
**19**	N-Phenylacetyl-Gly-Lys	**59**	L-Ornithine monohydrochloride
**20**	Asp-Phe methyl ester(aspartame)	**60**	Pyridoxamine dihydrochloride
**21**	Cadaverine dihydrochloride	**61**	Thiamine hydrochloride
**22**	Putrescine dihydrochloride	**62**	Nicotinamide
**23**	L-Glutamine	**63**	Blasticidine S hydrochloride
**24**	Glycine	**64**	Colistin sulfate salt

**25**	L-Alanine	**65**	L-citrulline
**26**	L-Tryptophan	**66**	L-Homoserine
**27**	L-Leucine	**67**	L-isoleucine
**28**	Streptomycin sulfate salt	**68**	Sulfacetamide
**29**	Tobramycin	**69**	Cephalexin
**30**	Ampicillin	**70**	7-Aminocephalosporanic acid
**31**	NAD	**71**	Guanosine
**32**	Thiamine pyrophosphate	**72**	α-Methyl-DL-serine
**33**	Adenosine-3′,5′-cyclic monophospha(cAMP)	**73**	Folic acid
**34**	Urea	**74**	Pterine
**35**	Allantoin	**75**	AICAR(5-Aminoimidazole-4-carboxamide-1-β-D-ribofuranosyl- 5′-monophosphate)
**36**	Tyramine	**76**	4-Aminobenzoic acid
**37**	Serotonin hydrochloride	**77**	N-acetyl isoleucine
**38**	5-Amino-4-imidazolecarboxamide hydrochloride	**78**	N-Acetyl-L-tryptophan
**39**	L-Glutathione oxidized	**79**	N1-Acetylspermine trihydrochloride
**40**	Acetyl-Ser-Asp-Lys-Pro	**80**	N alpha-Acetyl-L-glutamine

Table S2: Data collection and refinement statistics of SaSpeG. Statistics for the highest-resolution shell are shown in parentheses.

	**SaSpeG (5IX3)**	**SaSpeG spm (8FV0)**	**SaSpeG spm (8FV1)**
Resolution range (Å)	37.99-1.81 (1.875 -1.81)	24.86 - 2.65 (2.745-2.65)	24.95-2.951 (3.056-2.951)
Space group	P 6 2 2	I 2 2 2	P 21 3
Unit cell	107.95 107.95 65.23 90 90 120	68.098 119.951 145.522 90 90 90	161.683 161.683 161.683 90 90 90
Unique reflections	20348 (1948)	17700 (1729)	29846 (2792)
Multiplicity	16.6 (16.4)	7.5(7.8)	20.8(19.0)
Completeness (%)	97.06 (95.54)	99.9 (100.00)	99.75 (99.97)
Mean I/sigma(I)	13.19 (2.22)	10.5(2.4)	18.9(1.9)
Wilson B-factor	26.54	29.72	68.05
R-merge	0.02035 (0.2922)	0.141(0.819)	0.153(1.899)
Reflections used for R-free		871 (89)	1440 (122)
R-work	0.2208 (0.3032)	0.2240 (0.2867)	0.2249 (0.3543)
R-free	0.2560 (0.3400)	0.2709 (0.2519)	0.2439 (0.3737)
Number of non-hydrogen atoms	1522	4214	5633
macromolecules	1404	4172	5577
ligands	-	114	152
Protein residues	166	496	664
RMS(bonds)	0.008	0.002	0.002
RMS(angles)	1.11	0.42	0.48
Ramachandran favored (%)	99.00	96.73	95.88
Ramachandran allowed (%)	1.00	3.27	4.12
Ramachandran outliers (%)	0.00	0.00	0.00
Average B-factor	30.5	43.87	79.08
macromolecules	30.1	43.91	79.17
ligands	-	39.99	69.84

Table S3: Interface interactions within the dodecamer of the SaSpeG PDB ID 5IX3 crystal structure determined using PISA.

Interface I

H-bonds	Chain	Dist(Å)	Chain
1	A:ASN 72[ND2]	3.59	B:TYR 26[OH]
2	A:ASN 77[ND2]	2.89	B:GLN 143[O]
3	A:HIS 112[NE2]	2.67	B:GLU 142[OE2]
4	A:LYS 113[NZ]	2.85	B:LYS 141[O]
5	A:LYS 113[NZ]	2.62	B:GLN 143[OE1]
6	A:LEU 139[N]	2.92	B:GLU 137[O]
7	A:LYS 141[N]	2.79	B:GLU 137[OE2]
8	A:TYR 26[OH]	3.59	B:ASN 72[ND2]
9	A:GLU 137[O]	2.92	B:LEU 139[N]
10	A:GLU 137[OE2]	2.79	B:LYS 141[N]
11	A:LYS 141[O]	2.85	B:LYS 113[NZ]
12	A:GLU 142[OE2]	2.67	B:HIS 112[NE2]
13	A:GLN 143[O]	2.89	B:ASN 77[ND2]
14	A:GLN 143[OE1]	2.62	B:LYS 113[NZ]
**Salt bridge**			
1	A:HIS 112[NE2]	2.67	B:GLU 142[OE2]
2	A:GLU 142[OE2]	2.67	B:HIS 112[NE2]

Interface II

H-bonds	Chain	Dist(Å)	Chain
1	A:THR 36[OG1]	2.80	C:GLU 7[OE1]
2	A:LEU 35[N]	3.34	C:GLU 7[OE1]
3	A:THR 36[N]	2.97	C:GLU 7[OE1]
4	A:LEU 35[N]	3.08	C:GLU 7[OE2]
5	A:TYR 145[OH]	2.74	C:PHE 106[O]
6	A:GLY 148[N]	3.05	C:ASN 107[O]
7	A:LYS 147[N]	3.29	C:ASN 107[O]
8	A:THR 36[OG1]	3.18	C:TYR 8[N]
9	A:GLU 33[OE2]	3.10	C:ARG 52[NE]
10	A:GLU 33[OE1]	2.36	C:TYR 104[OH]
11	A:TYR 150[OH]	2.99	C:ASN 110[ND2]
**Salt bridge**			
1	A:GLU 33[OE1]	3.84	C:ARG 52[NE]
2	A:GLU 33[OE2]	3.10	C:ARG 52[NE]

Interface III

H-bonds	Chain	Dist(Å)	Chain
1	A:ARG 76[NH1]	2.64	D:TYR 73[O]
2	A:ASN 110[ND2]	3.03	D:ILE 74[O]
3	A:TYR 73[O]	2.64	D:ARG 76[NH1]
4	A:ILE 74[O]	3.03	D:ASN 110[ND2]
